# A Real-World Pharmacovigilance Study of the FDA Adverse Event Reporting System Events for Trametinib

**DOI:** 10.7759/cureus.67925

**Published:** 2024-08-27

**Authors:** Xinyue Zhang, Rongrong Li, Yanrong Li, Lu He, Encun Hou

**Affiliations:** 1 Oncology, Guangxi University of Chinese Medicine, Nanning, CHN; 2 Oncology, Ruikang Hospital, Guangxi University of Chinese Medicine, Nanning, CHN

**Keywords:** tyrosine kinase inhibitor, hypertension, fatigue, hypothyroidism, faers, adverse event, trametinib

## Abstract

Objective: This research investigates adverse drug events (ADEs) linked to trametinib, utilizing data from the FDA Adverse Event Reporting System (FAERS) database, covering the period from Q2 2013 to Q4 2023.

Methods: We gathered data on ADEs associated with trametinib from the second quarter of 2013 to the fourth quarter of 2023. After standardizing the data, we applied various analytical methods to quantify signals, including the reporting odds ratio (ROR), proportional reporting ratio (PRR), Bayesian Confidence Propagation for Neural Networks (BCPNN), and multi-item gamma Poisson shrinker (MGPS).

Results: In our examination of 2200 ADE reports with trametinib cited as the primary suspect, we identified 191 adverse reaction terms across 23 system organ classifications. The most frequently reported classifications were general disorders and administration site conditions, with 1254 cases (ROR 0.83, PRR 0.85, IC -0.23, EBGM 0.85), followed by neoplasms (benign, malignant, and unspecified, including cysts and polyps) with 802 cases (ROR 3.59, PRR 3.32, IC 1.73, EBGM 3.32), and investigations with 794 cases (ROR 1.74, PRR 1.66, IC 0.73, EBGM 1.66). Notably, this study also uncovered previously unlabeled adverse reactions such as cheilitis, lobular panniculitis, ulcerative keratitis, and stridor.

Conclusion: While trametinib provides therapeutic advantages, it is associated with several potential adverse reactions. It is crucial for healthcare providers to closely monitor patients for symptoms such as cheilitis, lobular panniculitis, ulcerative keratitis, stridor, and other adverse events (AEs) during treatment.

## Introduction

Trametinib is a reversible and selective inhibitor of MEK1 (mitogen-activated protein kinase kinase 1) and MEK2 (mitogen-activated protein kinase kinase 2) and is used in combination with dabrafenib, another BRAF inhibitor, for treating the same medical condition. The combination therapy of trametinib and dabrafenib has produced a more robust and sustained therapeutic response compared to using either agent alone, highlighting the synergy between these two drugs. The convenience of trametinib administration is enhanced by its oral availability, allowing patients to take the medication without the need for intravenous infusions. As an adenosine triphosphate (ATP)-noncompetitive inhibitor, trametinib specifically targets MEK1 and MEK2 without competing with ATP, which is essential for the kinase activity of these enzymes. This selective inhibition of MEK1 and MEK2 ensures that trametinib effectively blocks the MAPK/ERK signaling pathway while minimizing potential off-target effects. Extensive testing has confirmed trametinib’s high specificity for MEK1 and MEK2 compared to other kinases. Its selectivity was validated against a broad panel of over 180 kinases, including closely related kinases such as B-Raf and C-Raf. This rigorous validation process underscores trametinib’s precision in targeting the intended molecules, thereby enhancing its therapeutic index and safety profile. The high degree of specificity and selectivity makes trametinib a valuable agent in the arsenal of targeted therapies for cancer, offering patients a treatment option that is both effective and well-tolerated [1]. Trametinib is an orally available, selective, ATP-noncompetitive inhibitor of MEK1 and MEK2. Its specificity for MEK1/2 was confirmed against over 180 kinases, including B-Raf and C-Raf [2].

Trametinib received approval from the US FDA in May 2013 and from the European Medicines Agency in September 2013 for the treatment of metastatic melanoma with the V600E mutation. This approval marked a significant milestone in the management of this aggressive form of skin cancer. However, it soon became evident that while trametinib monotherapy was effective, its progression-free survival and response rates were lower than those achieved with BRAF inhibitors. BRAF inhibitors had already set a high standard in treating BRAF-mutated metastatic melanoma by directly targeting and inhibiting the mutated BRAF protein. Given the limited results of trametinib as a single agent, researchers explored the potential benefits of combining trametinib with a BRAF inhibitor such as dabrafenib. The rationale behind this combination therapy was to enhance the therapeutic effects by simultaneously targeting different points within the same signaling pathway. Trametinib inhibits MEK1/2, while dabrafenib targets the upstream BRAF V600E/K mutations. By using both drugs together, it was hypothesized that this approach could more effectively impede the pathway responsible for tumor growth and survival, thereby delaying disease progression. Recent clinical data have supported this hypothesis, demonstrating that the combination of trametinib and dabrafenib significantly improves overall survival and progression-free survival compared to standard therapies, such as vemurafenib monotherapy. Vemurafenib, another BRAF inhibitor, had been a standard treatment, but the combined regimen of trametinib and dabrafenib showed superior outcomes. Patients treated with the combination therapy experienced a longer duration of disease control and a greater reduction in tumor burden. These findings have established the trametinib and dabrafenib combination as a more effective treatment option for patients with BRAF-mutated metastatic melanoma, offering new hope for improved long-term outcomes. In conclusion, while trametinib monotherapy offered a new treatment avenue for metastatic melanoma, its combination with dabrafenib has proven to be a more potent strategy. This combined approach not only enhances progression-free survival but also improves overall survival, representing a significant advancement in the therapeutic landscape for this challenging disease. The synergy between trametinib and dabrafenib underscores the importance of combination therapies in oncology, paving the way for further innovations in targeted cancer treatments [3].

While trametinib shows promise in treating cancer, it is not without potential adverse reactions. The FDA Adverse Event Reporting System (FAERS) is crucial for gathering and analyzing adverse drug events (ADEs) related to drug use [4]. The data gathered through the FAERS are invaluable for evaluating both the safety and effectiveness of medications. By systematically collecting reports of ADEs, FAERS provides a rich database that serves as a foundation for comprehensive drug safety assessments. This particular study utilizes the wealth of data available from FAERS concerning trametinib, a medication used in cancer treatment, to conduct an in-depth analysis of its safety profile. To achieve a nuanced and robust understanding of trametinib’s safety, the study employs various signal quantification methods. These methods include disproportionality analysis techniques such as the reporting odds ratio (ROR), proportional reporting ratio (PRR), Bayesian Confidence Propagation Neural Network (BCPNN), and multi-item gamma Poisson shrinker (MGPS). Each of these methods offers unique strengths in detecting and quantifying safety signals, thereby providing a multifaceted view of the potential adverse effects associated with trametinib. By leveraging multiple analytical approaches, the study ensures that the findings are both comprehensive and reliable. The use of ROR and PRR helps identify specific AEs that occur disproportionately more frequently with trametinib compared to other drugs. BCPNN and MGPS further validate these findings by incorporating Bayesian statistics and advanced modeling techniques, respectively. This combined approach not only enhances the robustness of the analysis but also mitigates the limitations inherent in any single method. The insights gained from this study are crucial for healthcare providers and regulatory agencies as they make informed decisions about trametinib’s use in clinical practice. By providing detailed information on the frequency and severity of AEs, the study helps in developing targeted strategies to manage risks and improve patient outcomes. Furthermore, the comprehensive safety profile established through this analysis supports ongoing monitoring and post-marketing surveillance of trametinib, ensuring that its benefits continue to outweigh potential risks.

In conclusion, the FAERS database is an invaluable resource for assessing the safety of medications. This study’s use of FAERS data on trametinib, combined with various signal quantification methods, provides a detailed and reliable analysis of its safety profile.

## Materials and methods

Data source

In our study, trametinib cases were identified using both the “PROD_AI” (product active ingredient) and “DRUGNAM” (drug name) fields in the FAERS database. We considered various expressions of trametinib, including “TRAMETINIB DIMETHYL SULFOXIDE,” “DABRAFENIB\TRAMETINIB,” and “TRAMETINIB,” as well as the drug names such as “MEKINIST,” “GSK1120212,” “TAFINLAR COMBO,” and various dosage forms. This comprehensive approach ensured that we captured all relevant instances of trametinib administration. This study made extensive use of American Standard Code for Information Interchange (ASCII) report files sourced from the FAERS database [5]. The period covered in this research extends from the second quarter of 2013 through the fourth quarter of 2023. To facilitate a thorough analysis, the ASCII files were systematically imported into the R programming environment, which is well-suited for complex data processing and statistical analysis. In order to ensure that our methodology and findings are transparent and can be independently verified, detailed information about the data used, along with comprehensive data sets, are publicly available. Interested parties can access these resources via the FDA’s website at (https://fis.fda.gov/extensions/FPD-QDE-FAERS/FPD-QDE-FAERS.html). This accessibility is critical as it supports the reproducibility of our research findings, allowing other researchers to replicate our methods and verify our results. Furthermore, providing open access to our data underscores our commitment to contributing valuable insights to the scientific community, thus fostering further research and discussion in this area.

Data processing

Redundant entries were filtered out from the demographics database, keeping only the latest entry for each unique case ID, determined by the date. The study focused specifically on reports where trametinib was indicated as the primary drug linked to ADEs. To detect drug-related safety signals, the analysis employed four disproportionality methods: the ROR [6], PRR [7], BCPNN [8], and MGPS [9]. Each method has distinct advantages: ROR helps minimize bias in reports of infrequent events, PRR is valued for its specificity, BCPNN integrates data from various sources enhancing cross-validation, and MGPS is effective in identifying signals from rare occurrences. This multifaceted approach not only expands the scope of detection but also cross-validates findings to boost the reliability of identified safety signals, thus minimizing the risk of false positives. The methodologies adjust thresholds and variance to pinpoint uncommon adverse reactions. Utilizing 2x2 contingency tables, detailed in Table 1, with further calculations and specific threshold values shown in Table 2, the statistical analysis was carried out in R. A higher correlation value between trametinib and AEs, as reflected in the statistical analysis, indicates a more significant signal intensity. To ensure the integrity of our data for analysis, we standardized our dataset through a series of rigorous steps aimed at maintaining accuracy and consistency. We removed duplicate entries to prevent data inflation and ensure the accuracy of signal calculations. We standardized drug names and adverse event (AE) terms using the Medical Dictionary for Regulatory Activities (MedDRA), which facilitated consistent data handling and analysis. We excluded reports that did not meet our study’s criteria, such as those with incomplete or inconsistent data, to maintain the quality and reliability of our analysis. Data entries were strictly aligned with the study period, spanning from the second quarter of 2021 to the fourth quarter of 2023, to ensure temporal consistency in our analysis. These methodological choices and data standardization procedures underpin the robustness and reliability of our study’s findings, enabling a comprehensive evaluation of Tivozanib’s safety profile based on AE reporting.

**Table 1:** Four-grid table.

	Trametinib-related ADEs	Non-trametinib-related ADEs	Total
Trametinib	A	b	a + b
Non-trametinib	C	d	c + d
Total	a + c	b + d	N = a + b + c + d

**Table 2:** Four major algorithms used for signal detection. Equation- a: number of reports containing both the target drug and target adverse drug reaction; b: number of reports containing other adverse drug reactions of the target drug; c: number of reports containing the target adverse drug reaction of other drugs; d: number of reports containing other drugs and other adverse drug reactions. 95%CI: 95% confidence interval; N: the number of reports; χ^2^: chi-squared; IC: information component; IC025: the lower limit of 95% CI of the IC; E(IC): the IC expectations; V(IC): the variance of IC; EBGM: empirical Bayesian geometric mean; EBGM05: the lower limit of 95% CI of EBGM; ROR: reporting odds ratio; PRR: proportional reporting ratio; BCPNN: Bayesian Confidence Propagation Propensity for Neural Networks; MGPS: multi-item gamma Poisson shrinker.

Algorithms	Equation	Criteria
ROR	ROR=ad/b/c	lower limit of 95% CI>1, N≥3
	95%CI=e^ln(ROR)±1.96(1/a+1/b+1/c+1/d)^0.5^	
PRR	PRR=a(c+d)/c/(a+b)	PRR≥2, χ^2^≥4, N≥3
	χ^2^=[(ad-bc)^2](a+b+c+d)/[(a+b)(c+d)(a+c)(b+d)]	
BCPNN	IC=log_2_a(a+b+c+d)(a+c)(a+b)	IC025>0
	95%CI= E(IC) ± 2V(IC)^0.5	
MGPS	EBGM=a(a+b+c+d)/(a+c)/(a+b)	EBGM05>2
	95%CI=e^ln(EBGM)±1.96(1/a+1/b+1/c+1/d)^0.5^	

**Figure 1:**
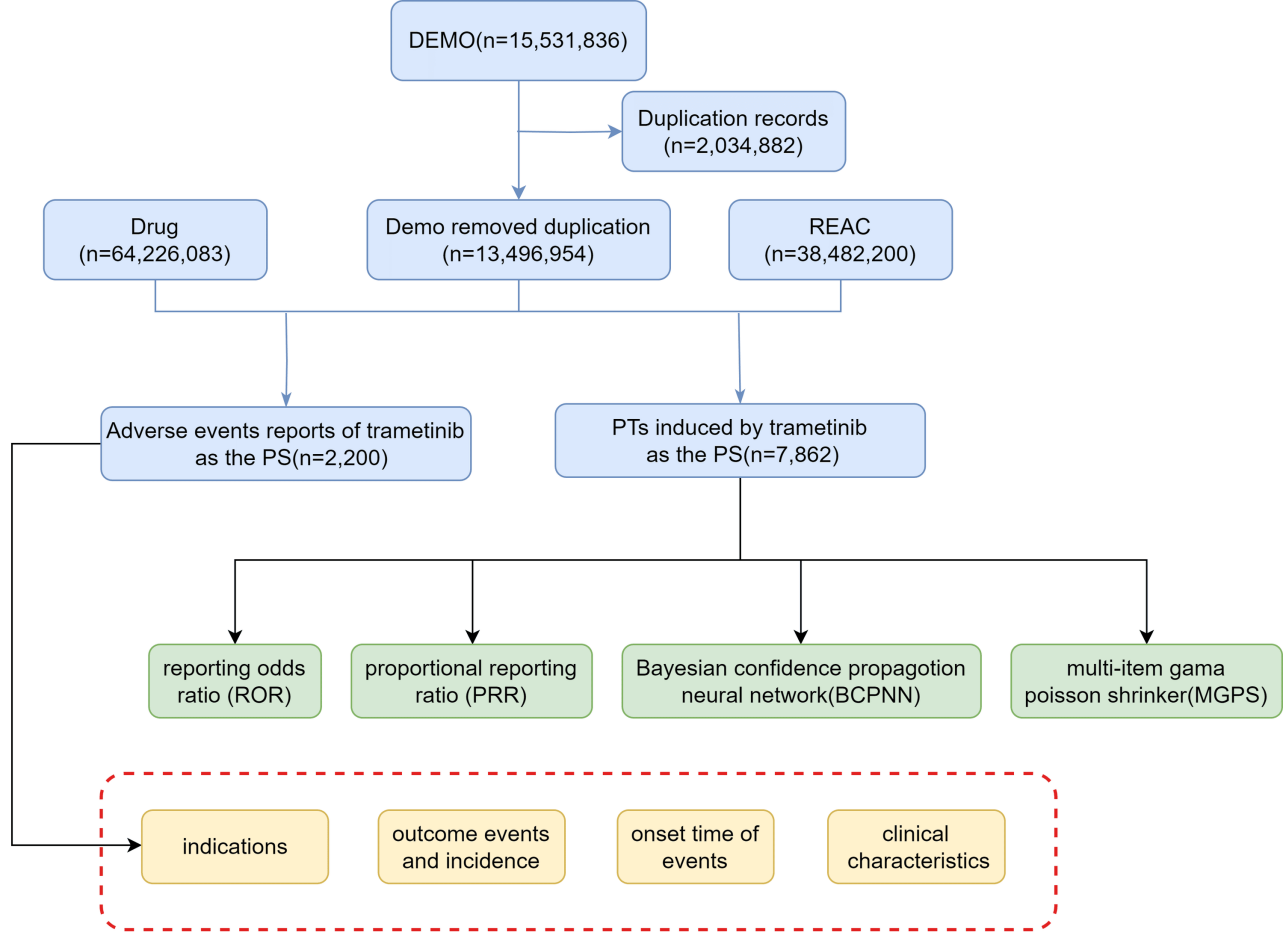
The flow diagram of selecting trametinib-related AEs from the FAERS database. PS: primary suspect drug; FAERS: The FDA Adverse Event Reporting System; REAC: **Reaction File** in the FDA Adverse Event Reporting System (FAERS), which lists adverse event reports. The number mentioned next to “REAC” (n=38,482,200) indicates the total number of adverse event instances reported in the database during the specified period.

Figure 1 depicts the methods used to associate trametinib with AEs in the FAERS database, illustrating the robust framework of this study.

## Results

Basic characteristics of trametinib-related AE reports

**Figure 2:**
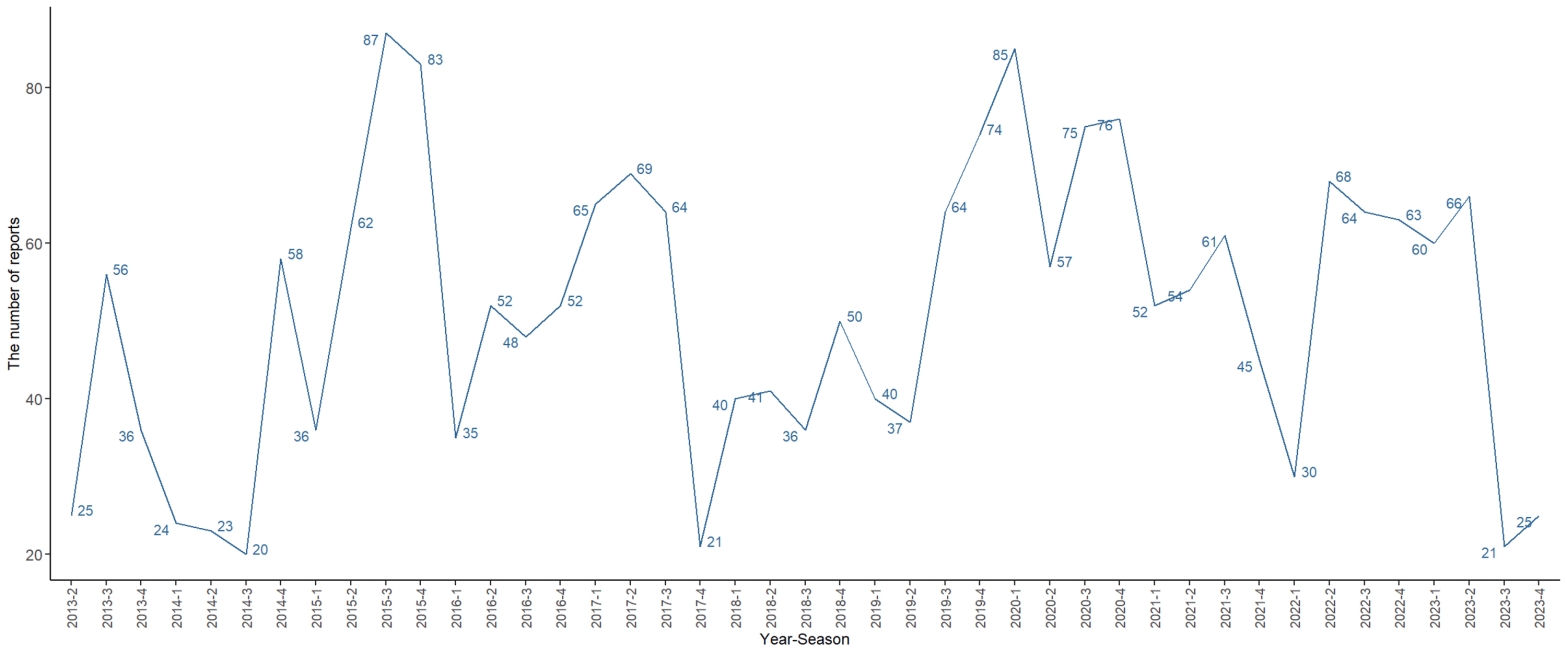
The number of AEs reported quarterly after the marketing of trametinib. AE: adverse effects

This study analyzed 15,531,836 AE reports from the FAERS database spanning from Q2 2013 to Q4 2023, with trametinib identified as the primary suspect in 2,200 of these reports, as illustrated in Figure 2. The data show an equal distribution of AEs between women (1,000 reports) and men (918 reports), though 12.82% of the reports lacked gender information, hindering a thorough gender-based analysis. The most represented age group was 45-65 years; however, 23.73% of the reports did not include age data, limiting the scope for age-specific analysis.

**Figure 3:**
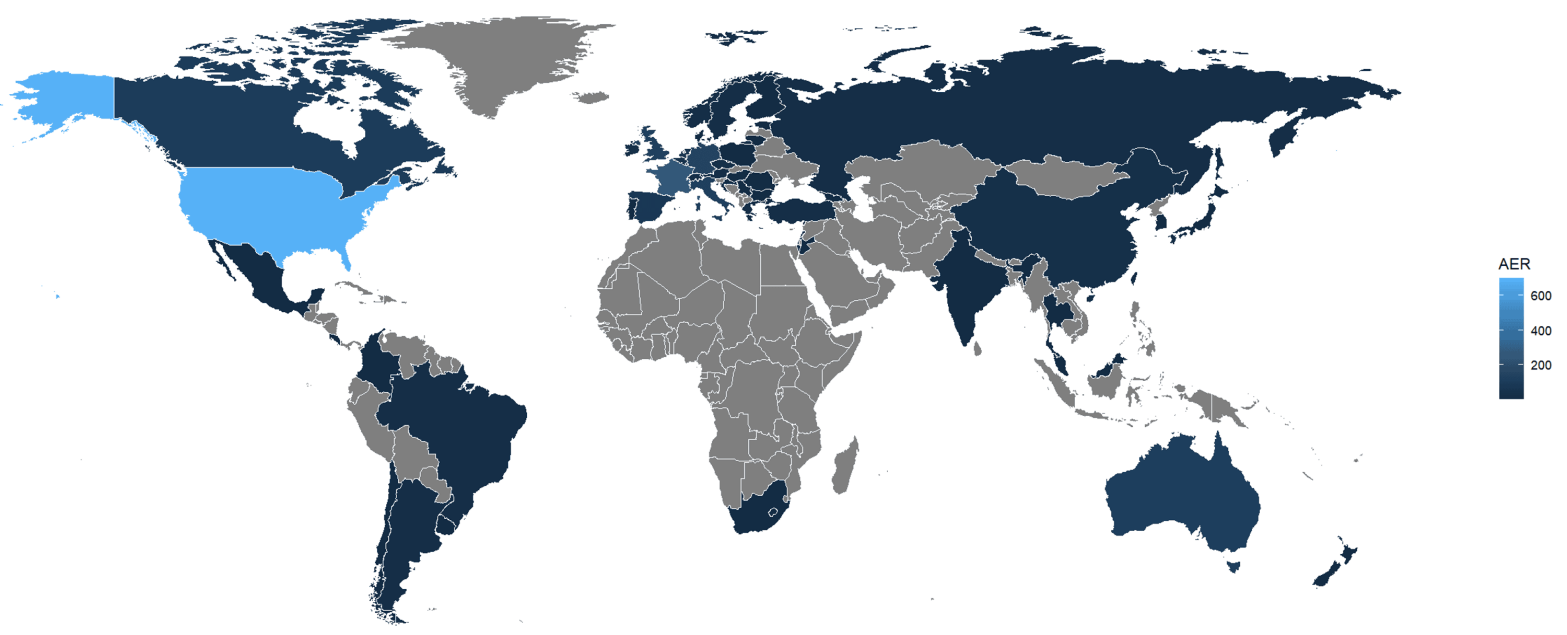
World distribution of the number of adverse reactions.

The majority of the reports were submitted by physicians (62.36%) and pharmacists (17.82%), with a relatively small percentage coming from consumers (5.18%), indicating minimal consumer participation in reporting. The bulk of the reports originated from the USA, as detailed in Figure 3. In terms of clinical outcomes, hospitalization was the most frequently reported AE (37.40%), with death being the next most common outcome (11.76%), as detailed in Table 3.

**Table 3:** Essential data regarding ADEs associated with trametinib extracted from the FAERS database. FAERS: The FDA Adverse Event Reporting System

Variable	Total
Year	
2013	117 (5.32)
2014	125 (5.68)
2015	268 (12.18)
2016	187 (8.50)
2017	219 (9.95)
2018	167 (7.59)
2019	215 (9.77)
2020	293 (13.32)
2021	212 (9.64)
2022	225 (10.23)
2023	172 (7.82)
Sex	
Female	1000 (45.45)
Male	918 (41.73)
Unknown	282 (12.82)
Age	
<18	295 (13.41)
18-45	251 (11.41)
45-65	599 (27.23)
65-75	341 (15.50)
>=75	192 (8.73)
Unknown	522 (23.73)
Reporter	
Physician	1372 (62.36)
Pharmacist	392 (17.82)
Other health professional	319 (14.50)
Consumer	114 (5.18)
Unknown	3 (0.14)
Reported countries	
United States	698 (31.73)
Unknown	639 (29.05)
France	250 (11.36)
Germany	148 (6.73)
United Kingdom	128 (5.82)
Italy	122 (5.55)
Australia	114 (5.18)
Canada	101 (4.59)
Route	
Oral	1520 (69.09)
Other	680 (30.91)
Outcomes	
Other serious	1277 (45.79)
Hospitalization	1043 (37.40)
Death	328 (11.76)
Life-threatening	98 (3.51)
Disability	39 (1.40)
Required intervention to prevent permanent impairment/damage	3( 0.11)
Congenital anomaly	1 ( 0.04)
Adverse event occurrence time - medication date (days)	
<7	95 (5.98)
7-28	353 (22.22)
28-60	312 (19.63)
>=60	605 (38.07)
Unknown	224 (14.10)

Signals detection based on system organ classes (SOC) levels

This study examined AE reports related to trametinib, identifying AEs across 23 system organ classes (SOCs). The data highlight that the most common SOCs are general disorders and administration site conditions (n=1,254, ROR 0.83, PRR 0.85, IC -0.23, and EBGM 0.85), neoplasms (benign, malignant, and unspecified, including cysts and polyps) (n=802, ROR 3.59, PRR 3.32, IC 1.73, EBGM 3.32), and investigations (n=794, ROR 1.74, PRR 1.66, IC 0.73, EBGM 1.66), reflecting trametinib’s use as an antineoplastic agent. Further information is available in Table 4.

**Table 4:** The signal strength of ADEs of trametinib at the SOC level in the FAERS database. SOC: system organ classes; ADE: adverse drug events; FAERS: FDA Adverse Event Reporting System

SOC	Case Reports	ROR (95% CI)	PRR (95% CI)	Chi sq	IC (IC025)	EBGM (EBGM05)
General disorders and administration site conditions	1254	0.83 (0.78-0.88)	0.85 (0.8-0.9)	38.56	-0.23 (-0.31)	0.85 (0.81)
Neoplasms benign, malignant, and unspecified (incl cysts and polyps)	802	3.59 (3.33-3.86)	3.32 (3.13-3.52)	1342.08	1.73 (1.63)	3.32 (3.12)
Investigations	794	1.74 (1.61-1.87)	1.66 (1.57-1.76)	223.17	0.73 (0.63)	1.66 (1.56)
Gastrointestinal disorders	790	1.16 (1.08-1.25)	1.15 (1.08-1.22)	16.02	0.2 (0.09)	1.15 (1.08)
Infections and infestations	673	1.58 (1.46-1.71)	1.53 (1.41-1.65)	131.18	0.61 (0.5)	1.53 (1.43)
Skin and subcutaneous tissue disorders	549	1.22 (1.12-1.33)	1.21 (1.12-1.31)	20.46	0.27 (0.15)	1.21 (1.12)
Nervous system disorders	397	0.58 (0.52-0.64)	0.6 (0.54-0.66)	116.61	-0.74 (-0.89)	0.6 (0.55)
Respiratory, thoracic, and mediastinal disorders	390	1.02 (0.92-1.13)	1.02 (0.92-1.13)	0.14	0.03 (-0.12)	1.02 (0.94)
Injury, poisoning, and procedural complications	375	0.42 (0.38-0.46)	0.45 (0.41-0.5)	287.82	-1.16 (-1.31)	0.45 (0.41)
Eye disorders	298	1.88 (1.67-2.11)	1.84 (1.64-2.07)	117.67	0.88 (0.72)	1.84 (1.67)
Blood and lymphatic system disorders	279	2.12 (1.88-2.39)	2.08 (1.85-2.34)	158.61	1.05 (0.88)	2.08 (1.88)
Metabolism and nutrition disorders	273	1.63 (1.44-1.84)	1.61 (1.43-1.81)	64.22	0.69 (0.51)	1.61 (1.45)
Cardiac disorders	232	1.24 (1.09-1.41)	1.23 (1.09-1.38)	10.49	0.3 (0.11)	1.23 (1.11)
Musculoskeletal and connective tissue disorders	184	0.41 (0.35-0.47)	0.42 (0.37-0.48)	153.87	-1.24 (-1.45)	0.42 (0.37)
Hepatobiliary disorders	137	2.05 (1.73-2.42)	2.03 (1.74-2.37)	72.07	1.02 (0.78)	2.03 (1.76)
Vascular disorders	132	0.79 (0.66-0.94)	0.79 (0.66-0.94)	7.44	-0.34 (-0.59)	0.79 (0.68)
Renal and urinary disorders	130	0.82 (0.69-0.98)	0.83 (0.7-0.99)	4.86	-0.28 (-0.52)	0.83 (0.71)
Psychiatric disorders	73	0.15 (0.12-0.19)	0.16 (0.13-0.2)	333.76	-2.62 (-2.95)	0.16 (0.13)
Endocrine disorders	30	1.44 (1.01-2.07)	1.44 (1.01-2.05)	4.06	0.53 (0.02)	1.44 (1.07)
Immune system disorders	25	0.26 (0.17-0.38)	0.26 (0.18-0.38)	53.38	-1.94 (-2.5)	0.26 (0.19)
Congenital, familial, and genetic disorders	18	0.75 (0.47-1.2)	0.75 (0.47-1.2)	1.45	-0.41 (-1.06)	0.75 (0.51)
Reproductive system and breast disorders	14	0.22 (0.13-0.36)	0.22 (0.13-0.37)	39.95	-2.21 (-2.94)	0.22 (0.14)
Ear and labyrinth disorders	13	0.36 (0.21-0.63)	0.36 (0.21-0.62)	14.46	-1.46 (-2.21)	0.36 (0.23)

Signals detection based on preferred term levels

**Figure 4:**
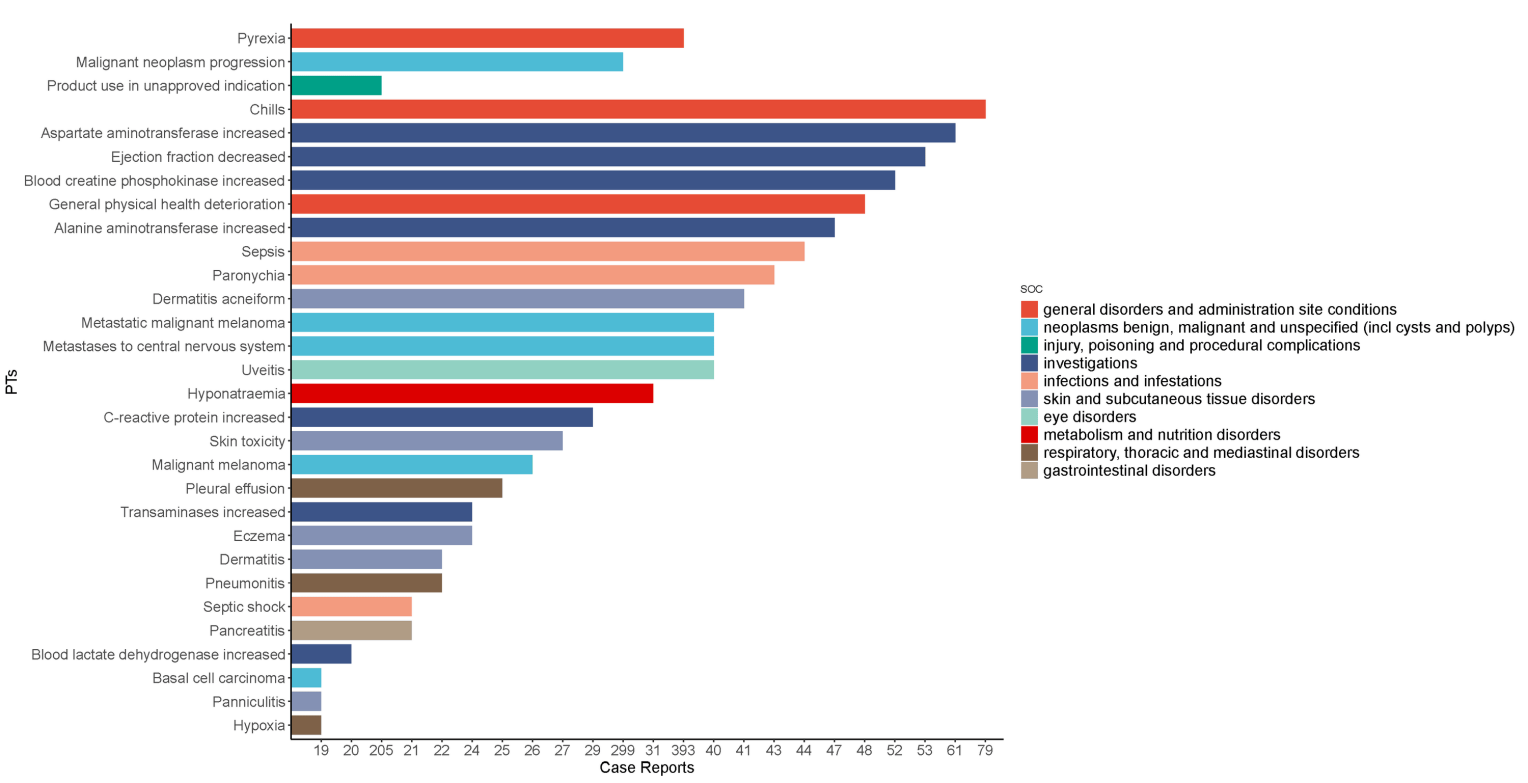
Bar graphs show the number of the top 30 AEs for trametinib in the FAERS database. The color indicates the SOC corresponding to the PT. The abscissa represents the number of reports. AE: adverse effects; FAERS: FAERS: The FDA Adverse Event Reporting System; SOC: System Organ Class

In this analysis, 191 preferred terms (PTs) were identified, with the most significant 15 PTs including pyrexia (n=393), malignant neoplasm progression (n=299), and product use in unapproved indications (n=205), among others, as detailed in Figure 4. Notably, the study uncovered previously unreported adverse reactions such as cheilitis, lobular panniculitis, ulcerative keratitis, and stridor. These new findings are important as they expand our understanding of trametinib’s safety profile. Cheilitis, characterized by lip inflammation, and lobular panniculitis, an inflammation of the subcutaneous fat, were significant observations. Reports also included ulcerative keratitis, a severe corneal condition, and stridor, a condition marked by a high-pitched wheezing sound. The identification of these adverse reactions highlights the critical need for ongoing surveillance and detailed reporting on drug safety. This information is vital for healthcare professionals in optimizing patient care with trametinib, ensuring thorough risk assessments, and making informed treatment decisions. Furthermore, these insights prompt further research into the causes of these reactions and the development of effective management strategies.

Onset time of events

**Figure 5:**
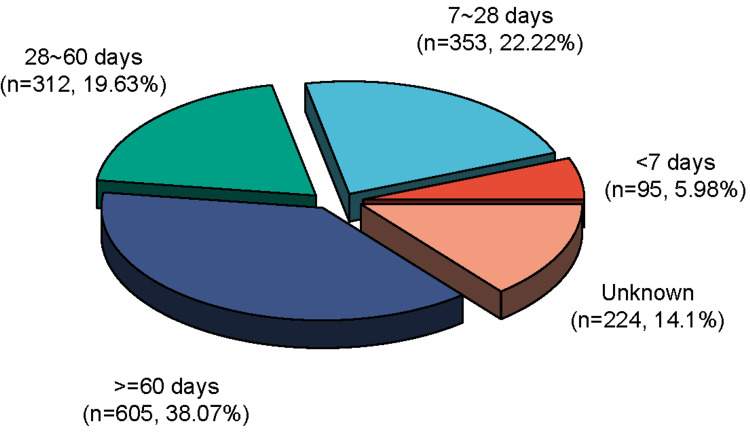
Time to onset of trametinib-related adverse AEs. AE: adverse effects

As shown in Figure 5, a significant majority of the cases - 448 instances or 28.2% - were reported within the first 28 days following the administration of trametinib. This concentration of cases in the initial four weeks underscores the importance of this period as a critical window for vigilant monitoring of patients to detect potential adverse reactions early. Specifically, the data shows that 312 cases (19.63%) occurred between 28- and 60 days post-administration. This indicates that while the likelihood of encountering adverse reactions diminishes over time, a considerable number still emerge during this later period, albeit at a reduced frequency compared to the initial days.

Post the 60-day mark, the frequency of reported adverse reactions decreased further, with 605 cases (38.07%) occurring after this period. This trend suggests that while early post-administration periods are crucial, monitoring should continue well beyond the initial months to capture all possible delayed adverse reactions. However, complicating this analysis is the fact that 14.10% of the AE reports did not include information on the timing of the reactions. This absence of data introduces a significant layer of uncertainty, limiting our ability to precisely determine when some AEs begin and highlighting a gap in the data collection process. The lack of timing information not only hampers our understanding of the exact onset of adverse reactions but also poses challenges in assessing the full scope of potential delayed reactions. This deficiency in data underscores the need for more robust and comprehensive reporting practices, ensuring that future analyses can rely on complete datasets to accurately evaluate and understand the temporal dynamics of adverse reactions.

## Discussion

Monitoring the real-world usage and AEs of a medication post-market release is crucial for ensuring its safety and efficacy. This study conducted a systematic investigation into the adverse effects associated with trametinib by thoroughly analyzing the FAERS database from the second quarter of 2013 to the fourth quarter of 2023. Through this meticulous examination, the research not only reaffirmed known safety information but also identified potential new risks. The findings derived from this comprehensive analysis provide healthcare professionals and policymakers with more detailed and accurate information, enabling them to make informed decisions to support medical practice and public health initiatives. Below, we delve into an in-depth analysis of the research outcomes, exploring the implications and significance of the findings in greater detail.

A notable limitation arises from a significant portion of the data lacking specific age details, which hampers our ability to comprehensively understand AE frequencies across different age groups. Thus, future research endeavors should prioritize obtaining precise age information and delve into exploring potential variations in drug reactions among diverse age demographics, enabling a more nuanced understanding of trametinib’s safety profile in different age cohorts. Moreover, it is noteworthy that only 5.18% of cases were reported by consumers, underscoring a concerning gap in physician reporting within the FAERS database. This emphasizes the importance of enhancing consumers’ engagement and awareness regarding the significance of reporting AEs, thereby ensuring a more comprehensive and accurate representation of AEs associated with trametinib and other medications. The highest number of reports came from the USA, with details provided in Figure 3. This observation necessitates further investigation to elucidate any underlying factors contributing to these regional disparities and ascertain whether regional or cultural biases may influence reporting practices. In conclusion, while the comparable incidence of AEs between genders provides reassurance regarding trametinib’s safety profile, addressing the limitations related to age data completeness and physician reporting is imperative for enhancing the accuracy and reliability of post-market surveillance efforts. Furthermore, investigating regional or cultural patterns in reporting practices will facilitate a more comprehensive understanding of the real-world safety profile of trametinib and inform targeted interventions to optimize patient safety.

MEK inhibitors and BRAF inhibitors display distinctly different profiles regarding skin toxicity. Notably, while 10% of individuals treated with dabrafenib experience cutaneous squamous cell carcinoma or keratoacanthoma, this incidence increases to around 18% with vemurafenib usage [10]. On the other hand, trametinib has not been associated with these types of skin cancers, marking a significant deviation in side effect profiles between these treatments. Trametinib is known to induce a papulopustular rash, which contrasts sharply with the hyperkeratotic maculopapular rash typically seen in patients treated with vemurafenib. Additionally, trametinib can provoke acneiform eruptions similar to those triggered by cetuximab, another cancer treatment targeting the epidermal growth factor receptor. These skin eruptions are predominantly found in areas with dense sebaceous glands, such as the face, chest, and back, and are typically managed with topical antibiotics [11]. The onset of skin reactions usually occurs soon after the initiation of trametinib treatment, which underscores the critical need for patients to have immediate access to medical professionals as these side effects can develop rapidly. Early intervention with monitoring and dose adjustments is crucial and can greatly enhance patient outcomes. By closely observing the patient’s response in the initial weeks, healthcare providers can mitigate severe reactions and establish an optimal dosing regimen within the first two months of therapy. Furthermore, the strategy for managing trametinib toxicity includes temporary discontinuation of the medication followed by a reduction in dose [12]. This approach allows for the careful management of side effects while maintaining the therapeutic integrity of the treatment. Beyond these common reactions, trametinib is also associated with a spectrum of rarer toxicities that are characteristic of the MEK inhibitor class. These include pyrexia, ophthalmic issues like retinal vein occlusion and uveitis, cardiac complications such as cardiomyopathy and decreased left ventricular ejection fraction, venous thromboembolism, interstitial lung disease, and gastrointestinal bleeding [4]. Such diverse potential side effects necessitate a comprehensive and attentive approach to patient care, ensuring that all potential risks are managed effectively to optimize treatment efficacy and patient safety.

The AEs associated with trametinib and their underlying mechanisms are currently not well understood and remain largely speculative. A thorough determination of these causal relationships necessitates comprehensive investigations, taking into account not only the pharmacological properties of the drug itself but also the considerable individual variability among patients. Additionally, pre-existing medical conditions play a significant role in how these AEs manifest, further complicating the understanding of their origins. As such, it is imperative that further clinical and experimental studies be conducted to gain a more detailed insight into the adverse effects associated with trametinib. In light of these complexities, the role of healthcare providers becomes crucial. Clinicians must be vigilant in monitoring the onset and progression of AEs in patients undergoing treatment with trametinib. This vigilant observation is essential to manage and mitigate these effects proactively. Effective management of AEs not only involves addressing symptoms as they arise but also adjusting therapeutic strategies in response to individual patient responses and tolerances. By doing so, clinicians can ensure that treatment efficacy is maximized while minimizing potential harm to the patient. Therefore, ongoing research, coupled with attentive clinical care, is key to optimizing the use of trametinib and improving patient outcomes in the context of its associated AEs.

In this study, we conducted a comprehensive and systematic analysis of the FAERS database to evaluate the post-marketing safety characteristics of trametinib. Although FAERS is a spontaneous reporting system with variability in the completeness and accuracy of information, this can lead to biases in data analysis. We noted that underreporting is a major issue due to the voluntary nature of the reports, and the reports in the database do not establish causality, only indicating potential AEs. Additionally, several unmeasured confounding factors, such as potential drug interactions, comorbidities, and concurrent medications, were not included in the study, adding complexity to the interpretation of the data. Despite these limitations, our research identified three unexpected and significant AEs, including Langerhans cell histiocytosis, endocarditis bacterial, and hypocapnia, thus expanding the safety information known from clinical trial phases. These findings provide valuable references for healthcare professionals to closely follow up with patients and monitor drug-related AEs in clinical practice. We recognize that while FAERS data provide preliminary signals about potential risks, determining the true incidence and causality requires further validation through prospective clinical trials. This study not only supplements the information in the FAERS rare AE system but also provides direction for future research, advancing our understanding of the safety of trametinib in real-world clinical settings.

## Conclusions

This study offers a comprehensive analysis of trametinib-related AEs by leveraging data from the FAERS database spanning from Q2 2013 to Q4 2023. Our findings reaffirm trametinib’s established safety profile. Importantly, this study revealed previously unlabeled adverse reactions such as cheilitis, panniculitis lobular, ulcerative keratitis, and stridor associated with trametinib use. This emphasizes the importance of vigilant post-marketing surveillance and extended monitoring periods for patients administered trametinib. Despite limitations such as incomplete data and potential reporting biases inherent to FAERS, this research provides valuable insights for healthcare professionals, highlighting the need for enhanced AE reporting and monitoring practices. Future studies should aim to validate these findings through prospective clinical trials and address regional disparities in reporting to further refine our understanding of trametinib’s safety in diverse patient populations. The identification of previously unreported AEs, emphasizes the continuous need for robust pharmacovigilance to ensure patient safety and inform clinical decision-making.
